# A Comprehensive Review of Myocardial Bridging: Exploring Diagnostic and Treatment Modalities

**DOI:** 10.7759/cureus.43132

**Published:** 2023-08-08

**Authors:** Endurance O Evbayekha, Enyioma Nwogwugwu, Adedoyin Olawoye, Kafayat Bolaji, Adeyemi A Adeosun, Abimbola O Ajibowo, G. Chinenye Nsofor, Vivian N Chukwuma, Hameed O Shittu, Chinwendu A Onuegbu, Adewale M Adedoyin, Okelue E Okobi

**Affiliations:** 1 Internal Medicine, St. Luke's Hospital, Chesterfield, USA; 2 Internal Medicine, Lincoln Medical and Mental Health Center, New York, USA; 3 Internal Medicine, Maimonides Medical Center, New York, USA; 4 Ophthalmology, Aminu Kano Teaching Hospital, Kano, NGA; 5 Molecular Pharmacology and Experimental Therapeutics, Mayo Clinic, Rochester, USA; 6 Internal Medicine, Lugansk Medical University, Lugansk, UKR; 7 Internal Medicine, Buckinghamshire Healthcare NHS Trust, Buckinghamshire, GBR; 8 Internal Medicine, University of Illinois at Chicago/Advocate Christ Medical Center, Chicago, USA; 9 Internal Medicine, Federal Medical Centre, Abeokuta, NGA; 10 Internal Medicine, Montefiore Medical Center, Wakefield Campus, Bronx, USA; 11 Internal Medicine, Lagos State University Teaching Hospital, Ikeja, NGA; 12 Family Medicine, Medficient Health Systems, Laurel, USA; 13 Family Medicine, Lakeside Medical Center, Belle Glade, USA

**Keywords:** myocardial bridging, interventions, imagining, modalities, atherosclerosis, angina, myocardial tunneling, myocardial bridge

## Abstract

Myocardial bridging (MB) is a congenital coronary artery anomaly involving an overlying myocardium's partial or complete encasement of a coronary artery segment. The obstruction can lead to significant cardiac symptoms, resulting in myocardial ischemia, arrhythmia, and sudden cardiac death. Several approaches, including invasive and non-invasive methods, have been proposed to diagnose and manage MB. Invasive modalities, such as intravascular ultrasound (IVUS) and coronary angiography, offer high specificity and sensitivity. In contrast, non-invasive methods like Doppler ultrasound, multislice computed tomography (MSCT), and magnetic resonance imaging (MRI) are advantageous due to their non-invasive nature, high sensitivity and specificity, and cost-effectiveness. Treatment options for MB mainly focus on relieving symptoms and preventing adverse outcomes. The use of pharmacological agents and surgical and percutaneous interventions has been documented in numerous studies. Studies conclude that MB is a treatable cardiac anomaly, and a combined approach of diagnosis, treatment, and follow-up is necessary to reduce the morbidity and mortality associated with this condition.

## Introduction and background

Myocardial bridging (MB) is a term used to describe the encroachment of the coronary artery past its natural epicardial domain and into the myocardium. It is regarded as an abnormal but benign variant, although it has been increasingly diagnosed recently due to increased and more advanced imaging modalities [1,2]. Henric Reyman first described the phenomenon of a tunneled segment of the coronary artery during an autopsy in 1732. At the time, it was considered a benign condition [1,3]. The coronary arteries' bridged segment is sometimes called a ‘tunneled’ segment [3].

Diagnosing MB involves a combination of clinical evaluation, non-invasive imaging techniques, and invasive coronary angiography. Symptoms commonly associated with MB include angina pectoris due to compression of the bridged segment during systole. Additionally, various diagnostic modalities, such as electrocardiography (EKG), stress testing, echocardiography, and cardiac computed tomography (CT) angiography, play pivotal roles in confirming the presence of myocardial bridging and assessing its clinical significance [4]. In terms of definitive treatment, managing MB depends on several factors, including the severity of symptoms, the associated cardiovascular risk factors, and the extent of myocardial ischemia. Conservative approaches, such as lifestyle modifications, pharmacotherapy, and risk factor management may be employed for patients with mild or asymptomatic MB [3-5]. However, more aggressive therapeutic interventions might be necessary for those with persistent symptoms or significant myocardial ischemia. Among the interventional techniques available, percutaneous coronary intervention (PCI) with stent placement and surgical myotomy are viable options for relieving coronary artery compression and improving blood flow. However, the decision regarding the treatment modality should be individualized, considering patient-specific factors, lesion characteristics, and associated comorbidities [6-9].

Our review is a valuable resource for clinicians, researchers, and healthcare professionals involved in diagnosing and managing MB. By integrating the current knowledge and evidence-based practices, this review aims to enhance clinical decision-making, improve patient outcomes, and stimulate further research in this intriguing field.

## Review

Method of literature search

**Figure 1:**
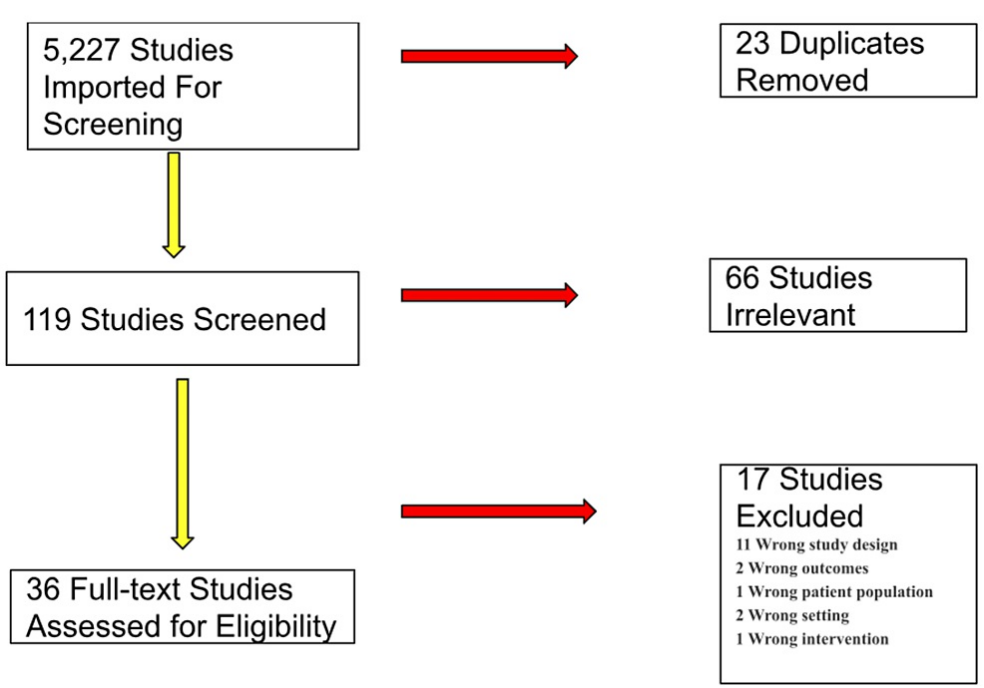
Methodology of systematic search

Our search and study selection was based on a defined set of inclusion and exclusion criteria. The search was conducted on PubMed, Google Scholar, and Cochrane Library. We included articles from the last 30 years (1993 to 2023) and considered randomized controlled trials, observation studies, reviews, systemic reviews, case series, and meta-analyses. We queried Google Scholar with the keywords 'myocardial bridge,' 'cardiovascular interventions,' 'myocardial bridging,' and 'cardiac tunneling.' We received a return of 16,800 results. We then set the timeline selection from 1993 to 2023 to give a 30-year timeframe, resulting in 11,800 articles. An advanced search with 'myocardial bridging' as the exact phrase found within the text in the literature resulted in 3,780 results. Then, we followed the same strategy on PubMed and found 1,315 results. We then filtered results by free full-text literature written in English over the last three decades (1993-2023) and found 283 results. We searched Cochrane Library and found 132 trials that contained our search words. This was not included in the final review due to a lack of access to the clinical trial databases. We assigned reviewer roles to five authors who screened the results by title and abstract to 119 matches. After reviewing the abstracts and considering our objectives, inclusion, and exclusion criteria, we narrowed them to 57 studies in full text. We analyzed 36 articles that directly documented the diagnosis and management of myocardial bridging, as highlighted in Figure 1.

Eligibility criteria

We reviewed various, original, full-text articles, randomized controlled trials, observation studies, reviews, systemic reviews, case series, and meta-analyses. Our inclusion timeline selection was from 1993 to 2023. We excluded non-English translated articles, articles without an available full text, those unrelated to MB and cardiac tunneling, and those outside the specified year range. The summary of our methodological process is in Figure 1.

Epidemiology

The incidence and prevalence per population vary widely based on the modality of diagnostic approaches within specific areas. Recent studies have shown a 25% prevalence with noninvasive modalities like coronary computed tomography. However, these percentages vary widely between 5% and 86% [1-3]; in one study, they were as high as 50% [3]. Invasive modalities do not appear to share the same reported findings, with a 1.7% (0.5-16%) prevalence [1,2], although a higher incidence was found in heart transplant recipients and individuals with hypertrophic cardiomyopathy. Some studies approximate 1-5% for symptomatic MB [1,4-6]. It may be challenging to determine the exact prevalence relying solely on invasive modalities, such as angiography, and this is because so many subjective variables, such as the observer's experience and intensity with which the bridging diagnosis is pursued, play a role. Also, the myocardial thickness, the tunneled vessels' length, and the myocardial fibers' orientation in relation to the coronary artery all contribute to the likelihood of the ease of diagnosing myocardial bridging with coronary angiography [1,4-6]. 

Pathophysiology

The spectrum of MB varies per person based on the characteristics of their coronary anatomy. Coronary angiography studies suggest that the left anterior descending coronary artery (LAD) is the most commonly involved vessel. In the majority, myocardial tunneling is of no clinical significance. However, when the vessel tunneling is buried in a significant position within the myocardium when it is affected by systolic cardiac contractions, it may pose various problems such as angina, stress cardiomyopathy, thrombosis, and infarction. Myocardial contraction may lead to the compression of the bridged segment causing a 'milking' phenomenon that may cause myocyte ischemia from decreased perfusion [7]. In a study done to examine the fractional flow reserve (FFR) in the bridged segment, there was a decrease in FFR from baseline and an exaggerated diastolic change when dobutamine infusion was administered after baseline measurements [7,8]. A few studies combined quantitative coronary angiography with Doppler flow measurements and intravascular ultrasound in individuals with tunneling of the isolated proximal left anterior descending coronary artery. Some observations were made regarding variations during the cardiac cycle, and they are the presence of a reduced diastolic to systolic velocity ratio, an almost pathognomonic systolic compression of the tunneled segment (maybe be concentric or eccentric) with relatively delayed dilation of the bridged segment during diastole; accelerated forward flow during early diastole, and a prominent peak in velocity occurring during this time [9,10].

Coronary artery spasm and MB

Coronary spasm, or coronary artery vasospasm, is a transient constriction of the coronary arteries, leading to reduced blood flow to the heart muscle. The exact mechanism is not fully understood but involves several factors. Endothelial dysfunction, where the inner lining of blood vessels fails to produce enough vasodilatory substances, may contribute to increased vasoconstriction. Hyperreactivity of smooth muscle cells in the artery walls can cause sudden and intense constriction. An imbalance in the autonomic nervous system, which controls blood vessel tone, may trigger excessive vasoconstriction. Certain substances released within the artery walls, such as endothelin-1 and thromboxane A2, possess vasoconstrictive properties. Hypersensitivity of the coronary arteries to various stimuli, like stress or certain drugs, may also induce spasms. Coronary spasms can occur in individuals with or without underlying artery narrowing. Diagnosis is typically made using coronary angiography with provocative drugs. Treatment involves medications that promote coronary artery dilation, such as calcium channel blockers, along with lifestyle modifications [9-14].

Coronary atherosclerosis and MB

**Figure 2:**
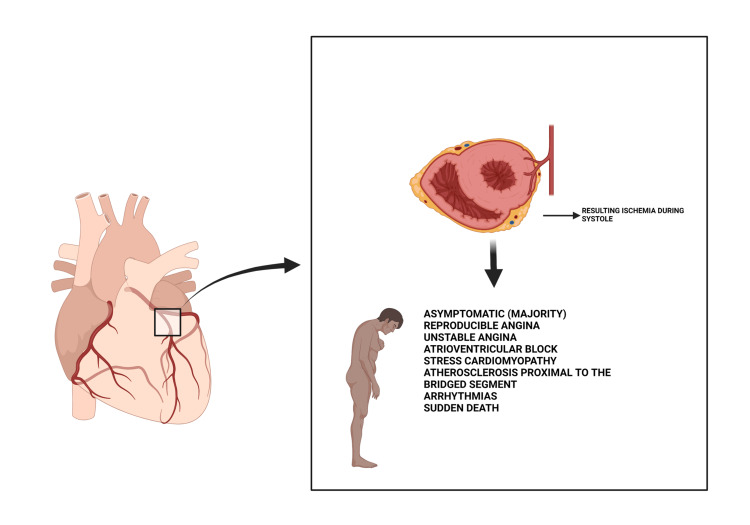
Anatomic abnormality of the coronary arteries with some possible presenting symptoms Original illustration created with Biorender.com

A few hypotheses suggested that MB might be protective in the development of atherosclerosis [11]. However, recent data show a positive correlation between MB and atherosclerosis. They postulate that mechanical shear forces during systole may propagate the progression of endothelial dysfunction leading to atherosclerosis in its characteristic pattern [12]. Studies of the blood flow through MB coronary arteries experience retrograde flow in systole [9]. This reason has been thought to be behind the development of atherosclerotic plaques immediately proximal to the MB [13,14]. This altered forward flow of blood through the vessel creates turbulence that causes undue mechanical force and results in an injury that may predispose to vessel dissection [15]. A few studies have reported concentric intimal thickening inferior to the bridged vessels [3,16]. Figure 2 highlights the possible presenting symptoms in MB patients.

Clinical presentation 

MB is most commonly an incidental finding because most patients are asymptomatic, a majority of whom are viewed as normal variants. For the minority with symptoms, they may present with symptoms of an acute coronary syndrome (ACS) such as reproducible, stable anginal pain, unstable angina, atrioventricular conduction block, Takotsubo syndrome, and rarely sudden death [13]. Some studies have shown that MB patients with ACS-like symptoms were younger, had higher tobacco use burdens, and lower traditional cardiovascular risk factors [17]. The electrocardiogram (EKG) is usually normal in asymptomatic patients with rare repolarization abnormalities seen during some stress tests [18]. The diagnosis is made when a substantial or entire coronary artery segment is embedded in the myocardium. The diagnosis may be made intraoperatively, during cardiac catheterization, coronary computed tomography, or postmortem examination.

Diagnostic modalities

Myocardial bridging has been a subject of interest since first detected in the 1960s after the introduction of invasive coronary angiography. Over time, multiple invasive and noninvasive techniques have been employed to diagnose myocardial bridging (MB). Still, there is no specific gold standard, and variabilities exist in the diagnostic accuracies of each method [13]. The fundamental anatomic and physiologic findings in MB are of epicardial coronary arteries coursing into the myocardium as explained above, and the effects of the myocardial contractility on the tunneled coronary arteries in systole and diastole and these are explored by each of these modalities of diagnosis. The initial classification of MB was based on the findings of invasive cardiac angiography based on the change in diameter between diastole and systole, called the "milking effect," but more recent noninvasive methods like computed tomography of the coronary arteries can demonstrate the intramyocardial course of the coronary arteries [19].

Noninvasive modalities include coronary computed tomographic angiography (CCTA); computed tomography fractional flow reserve (CTFFR); stress transthoracic echocardiography (TTE); stress myocardial perfusion imaging (MPI). Coronary CT angiography, CCTA, which is increasingly used to investigate chest pain syndromes, can visualize both the coronary artery lumen and the surrounding myocardium in MB. CCTA is also helpful in classifying MB as normal (epicardial), superficial, or deep [20]. For other imaging modalities like stress single-photon emission CT or positron emission tomography (PET) MRI, when combined with various stress protocols like physical exercise, adenosine, dobutamine, etc., it is possible to correlate ischemic symptoms to the degree of narrowing of the coronary vessels and perfusion defects. This is the basis for MPI [21].

CCTA can also derive fractional flow reserve (FFR) using computational fluid mechanics (CTFFR). However, this modality needs to be validated by more research and has limitations for CCTA and invasive FFR. Stress transthoracic echocardiography (TTE) is an indirect assessment of MB used to visualize the reversible wall motion hypokinetic changes associated with hypoperfusion. The best-studied perfusion region was the distribution of the LAD and a specific pattern of septal buckling with apical sparing is strongly correlated with MB. The invasive modalities are FFR, intracoronary Doppler; intracoronary wave-free ratio cardiac angiography (iFR), intravascular ultrasound (IVUS), and optical coherence tomography (OCT) [12,22].

Invasive modalities for MB are performed in catheterization labs and are used for a detailed assessment of the anatomy and physiology of tunneled coronary arteries. In a coronary angiogram, the milking effect of myocardial contractions produces a step-up and step-down appearance, which helps demarcate the affected segment of the coronary artery. A positive finding is when the intraluminal diameter is reduced by 70% or more during systole and persistently reduced by 35% or more during diastole. A pre-injection of vasodilators like nitroglycerin into the coronary artery can significantly improve diagnostic sensitivity [23]. Intravascular ultrasound involves introducing an ultrasonic tip into the angiogram wire and can produce a 3-D anatomical visualization of the size and morphology of the tunneled vessels. IVUS shows a characteristic half-moon echo-lucency due to a band of myocardium overlying the coronary vessel. However, IVUS cannot provide information about flow in the coronary artery [24]. Optical coherence tomography employs the principle of ultrasonography but emits light impulses on objects and senses the infrared waves from the objects to produce images. It has ten times the resolution of IVUS and can detect vulnerable plagues with better accuracy. It is also limited by the inability to evaluate flow changes [25].

Fractional flow reserves are a technique that measures the flow gradient proximal and distal to a fixed obstruction during the maximal flow (hyperemic) flow, which is usually achieved with the administration of adenosine. It is the gold standard in assessing fixed obstructions like atherosclerotic plaque but is limited in dynamic obstructions like MB. A difference between mean and diastolic FFR and dFFR provides a better assessment of MB. The ideal FFR is 1.0; a positive test will be FFR </=80% [26-27]. Instantaneous wave-free ratio (iFR) uses the principles of FFR but only in diastole. Compared to FFR, it is more sensitive in detecting obstructive bridges. A positive result is set at a </=89% cut-off for MB [28].

The spasm provocation test is a diagnostic procedure used to evaluate the impact of myocardial bridging on coronary artery vasospasm. A spasm-provoking substance like acetylcholine or ergonovine is injected into the coronary arteries during cardiac catheterization. Changes in blood flow and symptoms are monitored. The test helps determine if the tunneled segment of the coronary artery in myocardial bridging causes significant constriction and reduces blood flow during systole, leading to symptoms like chest pain (angina). It aids in confirming the role of myocardial bridging in inducing coronary spasms and guides appropriate management for symptomatic individuals [23-27].

Management

Pharmacotherapy

Patients with symptomatic MB often respond well to pharmacologic therapy in combination with antiplatelets considering their increased risk of atherosclerosis. Multi-slice CT scans could identify patients with sub-clinical atherosclerosis before initiating antiplatelet therapy [13]. Medical treatment aims to mitigate exacerbating factors that worsen MB such as hypertension, hypertrophy, tachycardia, reduced diastolic coronary filling time, and coronary artery compression [5]. Beta-blockers are considered first-line agents because of their negative chronotropic and inotropic effects, allowing for increased diastolic filling time, reduced compression of the tunneled coronary artery segment, and reduced sympathetic output [5,9]. Evidence of this was seen in an experimental study that showed a reversal of symptomatic ischemia after esmolol administration during atrial pacing with invasive hemodynamic assessments. Non-dihydropyridine calcium channel blockers are an alternative to beta-blockers in patients at risk of bronchospasm and are beneficial in patients with vasospasm [13]. However, vasodilatory agents should be avoided because of their increased risk of myocardial ischemia by dilating the proximal segments before the bridge, reducing the pressure gradient, and enhancing retrograde flow.

Percutaneous Intervention

Multiple studies have been carried out to evaluate the role and safety profile of PCI in the management of symptomatic myocardial bridging refractory to medical therapy. Several studies have reported high-target lesion revascularization rates at one year (24% of patients in one study and 36% in another) [26]. In addition, coronary perforation and stent fracture have also been reported in patients that underwent PCI. These complications could be related to histologic changes in the affected segments of these vessels, as described in one study that examined specimens from 45 cadavers [27]. Upon histologic evaluation of the proximal and tunneled segments of the affected vessel compared with a normal coronary artery, findings from this study highlighted a thin intima layer in the tunneled section compared with a hypertrophied intima in the proximal segment of the affected vessel. Changes in the epithelial cells were also noted. Several patients who underwent this procedure failed to achieve the desired outcome and continued to endorse symptoms. Therefore, PCI's usefulness for managing myocardial bridging refractory to medical management is limited and, therefore, not recommended.

Surgical Intervention

Surgical intervention involves either supra-arterial myotomy or coronary artery bypass graft (CABG). The heart muscle is meticulously and thoroughly dissected in a typical myotomy case. Wall perforation, ventricular aneurysm formation, and postoperative hemorrhage are all possible side effects of the myotomy. Contrarily, graft failure is the main issue with CABG regarding myocardial bridges. However, one series revealed an unintentional right ventricular wall perforation in 2/9 patients, and the other research stated that 1/26 patients had CABG for postoperative angina with LAD constriction. Two retrospective investigations on myotomy described overall effective operations. Regarding CABG, one analysis found no complications, whereas the second found that 15/39 patients had graft occlusions after follow-up and that 6/39 patients had recurrent angina. The authors concluded that grafting with the saphenous vein was better because grafting with the left internal mammary artery (LIMA) had a higher risk of occlusion than grafting with the saphenous vein (12 vs. 3 patients). This contrasts with an earlier study that suggested the LIMA as the ideal graft for CABG.

One research with 31 patients and a somewhat smaller series with 11 patients make up the studies contrasting the efficacy of myotomy and CABG in patients with symptomatic myocardial bridges. One myotomy case in the initial investigation underwent CABG following an unintentional right ventricular wall perforation. All 21/31 patients who underwent follow-up angiography (either myotomy or CABG) showed distal coronary blood flow restoration. In the second research, 2/11 participants with unusual chest pain received medical attention. Although myotomy and CABG are both viable initial options, which is better is not apparent. On the other hand, myotomy may be the preferred course of treatment for patients with symptoms of myocardial bridging resistant to medical therapy, angiographic evidence of coronary stenosis greater than 75%, or myocardial ischemia or infarction. This is because myotomy aims to treat the underlying pathology. However, CABG is preferred over myotomy when the myocardial bridge is large (more than 25 mm) or deep (greater than 5 mm) or when the spanned coronary segment fails to completely decompress in diastole (myotomy is unlikely to resolve the ongoing diastolic compression) [4,8]. It is significant because no randomized clinical trials contrast surgical surgery with an intensification of medicinal therapy. These sparse data imply that surgical treatment-myotomy or CABG appears safe and successful in symptomatic patients with myocardial bridging who are refractory to medications.

Comparing treatment options

**Figure 3:**
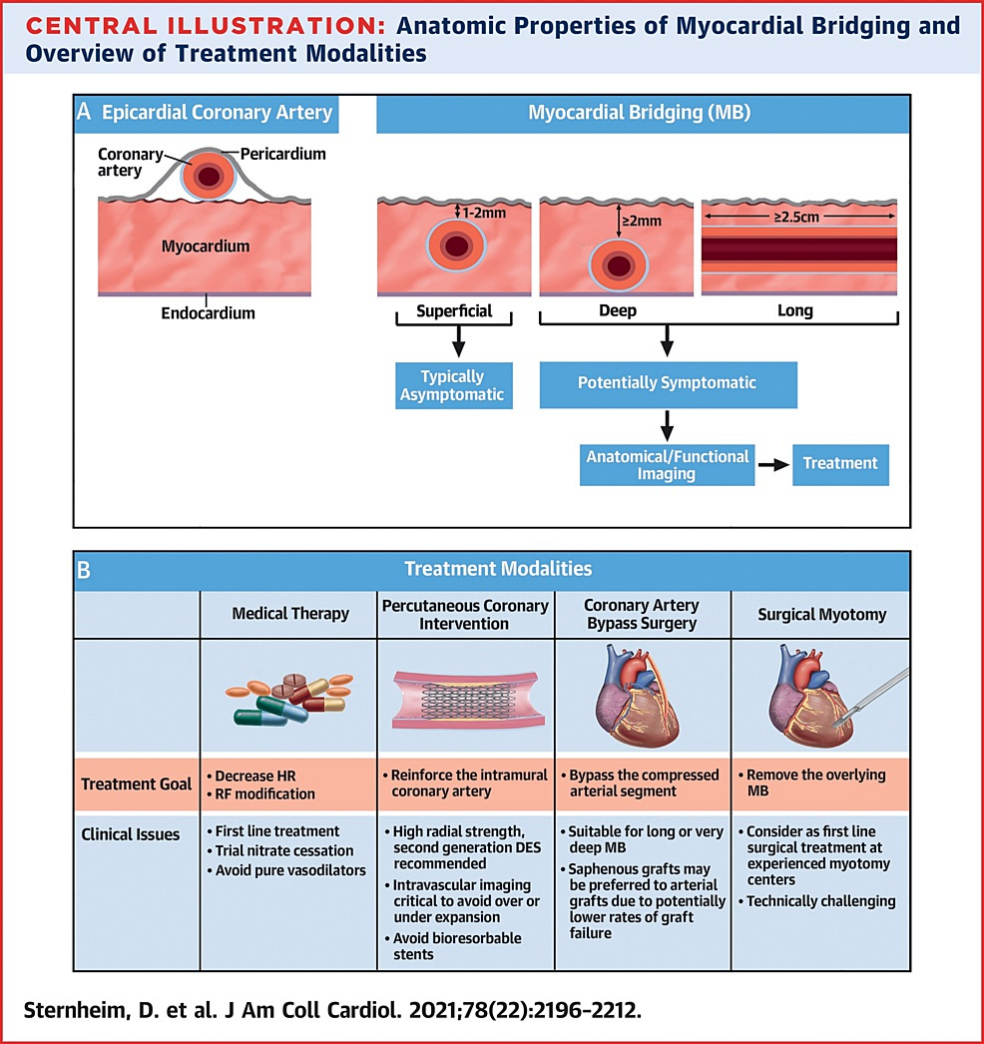
Classification and treatment modalities of myocardial bridging in recent years Illustration re-used with permission from jacc.org [19] MB = myocardial bridge; HR = heart rate; RF = risk factor

There are several factors to consider when choosing a therapeutic approach for managing myocardial bridging; presence and severity of symptoms, cardiac anatomy, including the characteristics of the bridged vessels, and possible complications, especially when invasive options are considered. It is important to note that although patients with MB often present with ischemic-type pain, nitrates are contraindicated in these patients due to the potential worsening of ischemia. Sternheim and colleagues developed the most widely accepted classification that has guided the treatment of MB in recent years, as depicted in Figure 3 [19]. A study by Hongo et al. found that nitrates caused increased narrowing of the bridged vessels due to the positive inotropic and chronotropic effect on the myocardium and increased vessel compliance [23].

Although beta-blockers and other negative inotropes are considered the first-line pharmacologic treatment for most patients with typical or atypical anginal pain related to MB, the effects of these drug therapies are often short-lived [20]. In addition, there is a limitation in the availability of pharmacologic treatment modalities for MB due to a poor understanding of its pathophysiology. Despite lacking a universal guideline for managing MB, most clinicians agree that surgical treatment should be pursued in patients whose symptoms are severe or unresponsive to pharmacologic therapy. In the simplified Table 1, clinicians may find this helpful during the rapid evaluation of patients with MB and help stratify them according to their class based on symptoms and clinical examination.

**Table 1:** A simple classification system that informs clinical decision-making

	Symptoms	Signs of ischemia	1st line treatment	2^nd^ line treatment
Type A	Present	Absent	Lifestyle modifications	
Type B	Present	Present	Beta-blockers or calcium channel blockers.	Invasive testing and PCI or surgery
Type C	Present	Present	Beta-blockers or calcium channel blockers.	PCI or surgery

When comparing the surgical options available, myocardial unroofing vs. CABG, some studies showed no significant difference in outcomes and minimal risk of complications, suggesting that both could be used in patients with refractory ischemic pain and the choice is dependent mainly on the clinicians' expertise [23]. However, in a study by Hemmati and colleagues, more than 60% of patients reported postoperative chest pain three years after a myocardial unroofing procedure, despite the short-term relief of symptoms. Their report also showed that many patients who underwent this surgical procedure still received pharmacotherapy with beta-blockers or non-dihydropyridine calcium channel blockers [29]. Despite these limitations, surgical treatment is preferred over stenting due to the increased revascularization rate within the first year [30]. Overall, there is a need for further prospective trials to evaluate the long-term outcomes of these treatment modalities.

Future trends in MB

Several recent advances have been made in diagnosing and managing myocardial bridging. Some of the potential future advancements in this area include: 1. Imaging modalities: advancements in imaging techniques, such as coronary CT angiography, can now produce high-resolution images of the coronary artery system and aid in detecting myocardial bridging. Other imaging techniques to determine the anatomic significance of myocardial bridging include cardiac magnetic resonance imaging, optical coherence tomography (OCT), Doppler flow wire (DFW), pressure wire approaches, positron emission tomography (PET) scan, and contrast stress echocardiography [13,31]. The hemodynamic significance of myocardial bridging can be established using intravascular ultrasound (IVUS), angiographic standards, or a dobutamine challenge to calculate the dFFR [32]. In addition to being frequently used to assess myocardial ischemia in people with known or suspected coronary artery disease, myocardial perfusion imaging (MPI) is also frequently used to determine the hemodynamic significance of MB [17]. 2. Risk stratification: clinicians may use risk stratification tools to identify myocardial bridging patients who are more likely to be at risk for complications and who may benefit from more aggressive treatment. 3. Medical therapy: Pharmacological treatment with beta-blockers is the first line of treatment for symptomatic MB to lower heart rate and decrease arterial compression induced by the muscular band, increasing the diastolic period. Calcium channel blockers are alternative medical therapy [32]. Future research may identify new pharmacological agents that can better manage the condition. When medical management fails, surgical intervention is warranted. 4. Interventional strategies: MB can be treated by creating individualized treatment programs suited to each patient's needs, considering the patient's particular physiology, symptoms, and risk factors. Interventional methods, such as stenting or surgical revascularization, may be required in patients with severe symptoms or problems related to myocardial bridging.

However, these strategies come with hazards, and each must be carefully evaluated [33]. Although CABG has been made available, it is not recommended due to the significant risk of graft failure [34]. However, when combined with unroofing, CABG may be a better option for patients with concurrent obstructive coronary artery disease (CAD) [32]. Coronary stenting is reserved for patients with refractory symptoms on medical management who are high-risk candidates for surgery. Unfortunately, high rates of stent fracture, in-stent restenosis, and coronary aneurysm have been reported [35]. Surgical unroofing of the MB resulted in dramatic symptomatic improvement and was shown to be a safe and effective therapeutic option, making it a more suitable and long-lasting remedy [32]. In a retrospective review, surgical unroofing for severe MB led to all postoperative patients becoming asymptomatic, with an improvement in New York Heart Association (NYHA) class from I-III to I-II [36]. 5. Long-term follow-up: Long-term follow-up of patients with myocardial bridging is necessary to monitor for complications and ensure effective management strategies. Future research may help identify optimal follow-up strategies for these patients. Overall, future trends in the diagnosis and management of myocardial bridging will likely focus on improving risk stratification, identifying new medical therapies, and optimizing interventional approaches while minimizing patient risks.

## Conclusions

Although mostly benign, a small percentage of symptomatic MB, If left untreated, is associated with significant morbidity and mortality. The diagnosis of MB requires a combination of invasive and non-invasive modalities, with non-invasive methods being preferred due to their less invasive nature and higher accuracy. Treatment of MB primarily focuses on relieving symptoms, reducing myocardial ischemia, and preventing adverse outcomes. Pharmacological agents are effective in milder cases while surgical and percutaneous interventions have been documented in severe cases. Combining an appropriate diagnostic modality with effective treatment and long-term follow-up is necessary to reduce the morbidity and mortality associated with MB. Further research is required to determine this condition's optimal diagnostic and treatment modalities.
